# Serum-Induced Proliferation of Human Cardiac Stem Cells Is Modulated via TGFβRI/II and SMAD2/3

**DOI:** 10.3390/ijms25020959

**Published:** 2024-01-12

**Authors:** Kazuko E. Schmidt, Anna L. Höving, Sina Kiani Zahrani, Katerina Trevlopoulou, Barbara Kaltschmidt, Cornelius Knabbe, Christian Kaltschmidt

**Affiliations:** 1 Department of Cell Biology, Faculty of Biology, University of Bielefeld, 33615 Bielefeld, Germany; k.schmidt10@uni-bielefeld.de (K.E.S.); sina.kiani@uni-bielefeld.de (S.K.Z.); katerina.trevlopoulou@uni-bielefeld.de (K.T.); barbara.kaltschmidt@uni-bielefeld.de (B.K.); c.kaltschmidt@uni-bielefeld.de (C.K.); 2 Institute for Laboratory and Transfusion Medicine, Heart and Diabetes Centre NRW, Ruhr-University Bochum, 32545 Bad Oeynhausen, Germany; 3 Medical Faculty OWL, University of Bielefeld, 33615 Bielefeld, Germany; 4 AG Molecular Neurobiology, Faculty of Biology, Bielefeld University, 33615 Bielefeld, Germany

**Keywords:** human cardiac stem cells, human blood serum, proliferation, senescence, ageing, RNA-Seq, TGFβ1

## Abstract

The ageing phenotype is strongly driven by the exhaustion of adult stem cells (ASCs) and the accumulation of senescent cells. Cardiovascular diseases (CVDs) and heart failure (HF) are strongly linked to the ageing phenotype and are the leading cause of death. As the human heart is considered as an organ with low regenerative capacity, treatments targeting the rejuvenation of human cardiac stem cells (hCSCs) are of great interest. In this study, the beneficial effects of human blood serum on proliferation and senescence of hCSCs have been investigated at the molecular level. We show the induction of a proliferation-related gene expression response by human blood serum at the mRNA level. The concurrent differential expression of the TGFβ target and inhibitor genes indicates the participation of TGFβ signalling in this context. Surprisingly, the application of TGFβ1 as well as the inhibition of TGFβ type I and type II receptor (TGFβRI/II) signalling strongly increased the proliferation of hCSCs. Likewise, both human blood serum and TGFβ1 reduced the senescence in hCSCs. The protective effect of serum on senescence in hCSCs was enhanced by simultaneous TGFβRI/II inhibition. These results strongly indicate a dual role of TGFβ signalling in terms of the serum-mediated effects on hCSCs. Further analysis via RNA sequencing (RNA-Seq) revealed the participation of Ras-inactivating genes wherefore a prevention of hyperproliferation upon serum-treatment in hCSCs via TGFβ signalling and Ras-induced senescence is suggested. These insights may improve treatments of heart failure in the future.

## 1. Introduction

During life the adult body requires mechanisms to renew organs and tissues as well as for damage repair after injury [1]. These regenerative processes are driven by ASCs which remain quiescent in their niche until the need for proliferation or differentiation due to injury, disease, or normal turnover of the cells [2,3,4]. These regenerative and homeostatic capacities of the body decline during the ageing process, which is defined as a progressive loss of physiological integrity leading to an increased vulnerability to age-related diseases [1,5]. In 2013, López-Otín and colleagues defined eight hallmarks of ageing including the exhaustion of stem cells and the increase of senescent cells, which both strongly contribute to the decline of the regenerative potential of the body [1]. The exhaustion of stem cells is mainly characterized by the accumulation of DNA-damage, expression of cell cycle inhibitory proteins and an overall decrease of cell cycle activity leading to reduced proliferation [1]. Accordingly, the investigation of stem cell proliferation at the molecular level is of great interest to counteract age-related diseases and increase the relative health span during life. Hence, the development of new cell-based methods and stem cell application is a fast-growing field in regenerative medicine. To this day, the treatments of bone marrow failure, muscular dystrophy or neurogenerative diseases as well as the support of myocardial regeneration have been reported in this context so far [6,7,8].

Notably, CVDs are the leading cause of death worldwide and are strongly linked to the ageing phenotype. CVDs occur along with decreased proliferation of resident cardiomyocytes and decreased regeneration potential of cardiac stem cells (CSCs) [9,10] which makes the development of rejuvenating stem cell-based strategies a promising approach to enhance the clinical outcome of CVD treatments. In terms of regeneration, particularly the adult human heart was long considered as an organ with low regenerative capacity until clonogenic and multipotent cardiac stem cells with the ability to self-renew were identified [6,11,12,13]. In previous studies we successfully isolated a Nestin^+^/S100^+^/c-kit^−^ cell population from human left atrial appendages (LAA) which expresses the cardiac progenitor markers CD105, CD31 and Sca-1. This stem cell population, namely human cardiac stem cells (hCSCs), was able to form cardiospheres after clonal growth and was shown to successfully differentiate into cardiomyocytes in vitro. In general, the transplantation of CSCs has already been shown to enhance the functional outcome for cardiac regeneration after heart failure in animals [14], which underlines the high medical applicability of stem-cell-based approaches for treatment of CVDs. However, studies targeting the activation of endogenous cardiac stem cells in order to overcome the need for surgical intervention were only carried out at a very basal level so far. Interestingly, young blood and plasma have been reported to have rejuvenating effects by several studies. For instance, the exposure of old mice to the blood of young mice by heterochronic parabiosis led to elevated regeneration of the muscle, the liver, the brain, the spinal cord, the olfactory system, and the heart [15,16,17]. Additionally, beneficial effects of young blood, plasma or serum in old tissues and organs as well as in various stem cell populations were described [16,18]. We could successfully underline these effects by showing serum-mediated proliferation and senescence reduction in hCSCs after application of human blood serum in vitro [11,19]. However, for the evaluation of a potential clinical application of young blood or its components, a deeper understanding of the molecular signalling networks is crucial. At a molecular level, the p38-MAPK/HSP27 axis was identified as a mediator of the reported serum effects in terms of hCSC migration, proliferation and senescence [19]. To fully elucidate the molecular mechanisms of these effects, further analysis of upstream or downstream pathways is necessary. In this association, the investigation of the TGFβ signalling pathway as a potential upstream mediator of the serum-induced effects is of great interest as it is known to be involved in proliferation and senescence [20,21,22,23,24,25].

TGFβ family members include several cytokines such as bone morphogenic molecules (BMPs), growth differentiation factors (GDFs), activins, Nodal and TGFβs occurring in three isoforms (TGFβ 1-3) [26]. All isoforms are translated with a large amino-terminal prodomain, which is necessary for correct folding and dimerization [27]. The latent form of TGFβ with furin cleaved but associated with the prodomain is also found in serum [28]. Activation of latent TGFβ1 can occur through interaction with extracellular matrix and α(v)β(6) integrin [29]. These ligands bind to the serine and threonine kinase receptors type II (TβRII) and type I (TβRI) on the outer cell membrane by building a receptor heterocomplex. Binding of a ligand to the homodimeric TβRII initiates conformational adaptation of the ligand and TβRII creates a new binding site with high affinity for TβRI. After recruitment of two TβRI units, the receptor heterocomplex is formed and the TβRII phosphorylates the serine residues of TβRI. During canonical SMAD signalling, the receptor-activated (R-) SMADs are subsequently phosphorylated by TβRI leading to downstream signal transmission [26]. Besides, induction of the TGFβ pathway also happens to activate non-SMAD signalling which comprises context-dependent induction of several pathways such as ERK-MAPK, p39/JNK, NF-κB, PI3K/AKT/mTOR, Rho-GTPase, JAK/STAT and p38-MAPK signalling [26]. In the heart in particular, the TGFβ signalling pathway plays an important role, as it is known to contribute significantly to cardiac development and vascularization [30,31,32,33,34,35,36]. Moreover, TGFβ1 is secreted by cardiac fibroblasts and cardiomyocytes thereby inducing myofibroblast differentiation [37,38]. Overall, these studies show that the investigation of this pathway is important in the context of cardiac regeneration.

In the present study, a proliferation-related response of hCSCs to serum treatment at the gene expression level was revealed by Gene Ontology. Additionally, the involvement of the TGFβ signalling pathway was indicated by differential gene expression analysis. Within this study, we demonstrate a dual role of the TGFβ signalling pathway in terms of proliferation and senescence in hCSCs. Differential gene expression analysis showed significant upregulation of proliferation-related and Ras-inactivating genes in hCSCs treated with serum and the TGFβRI/II inhibitor, compared to cells treated with serum alone. Our data suggest a prevention of hyperproliferation upon serum treatment in hCSCs via TGFβ signalling and Ras-induced senescence.

## 2. Results

### 2.1. RNA-Seq Analysis Reveals the Enrichment of Proliferation-Related Processes after Serum Treatment in hCSCs

**Figure 1:**
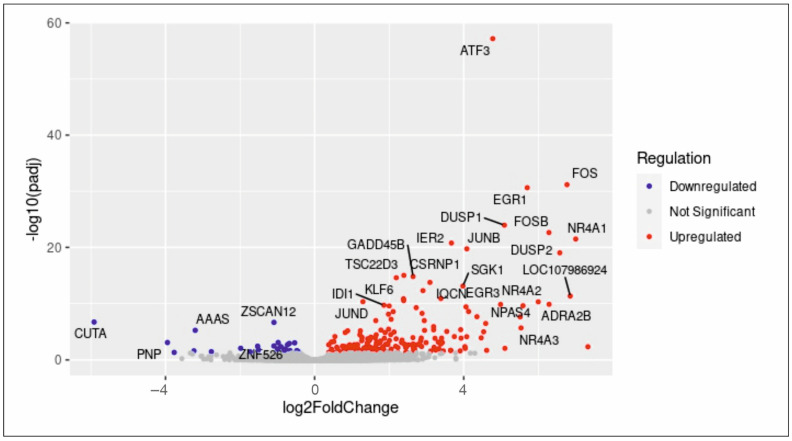
Human blood serum induces differential gene expression. Volcano plot shows upregulation of 213 differentially expressed genes after serum treatment and downregulation of 31 genes.

**Figure 2:**
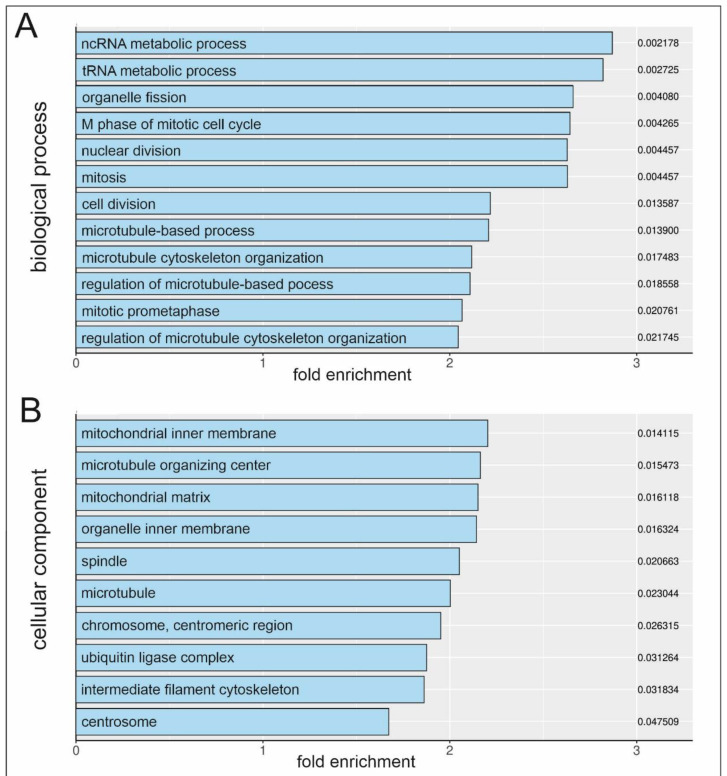

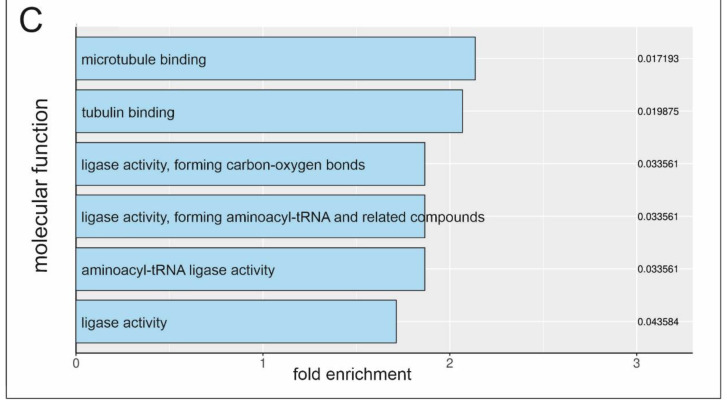
Human blood serum induces proliferation-related GO term enrichment. Upregulated GO terms regarding to (**A**) biological process, (**B**) cellular component and (**C**) molecular function.

To clarify the molecular mechanism underlying human blood serum-mediated proliferation in hCSCs, RNA sequencing (RNA-Seq) was performed. We previously published a proliferative and senescence reducing effect of human blood serum and compared differential gene expression after induction with human blood serum from old and young donors, revealing the p38-MAPK signalling pathway to be crucial for serum-mediated proliferation in hCSCs [11]. Here, the application of human blood serum resulted in the significant upregulation of 213 differential expressed genes and downregulation of 31 genes which underline the inductive effect of human blood serum on gene expression in hCSCs (Figure 1). Interestingly, the TGFβ signalling target gene ATF3 [39] and the endogenous TGFβ inhibitor NR4A1 [40] were differentially expressed after serum-treatment. The here observed upregulation of both targets and inhibitors of the TGFβ pathway indicate a crucial role of TGFβ in regulating the serum-mediated effects on hCSCs at the molecular level. GO term analysis between untreated and serum-treated hCSCs likewise showed a significant enrichment of several biological processes (BP) which are strongly related to proliferation. These comprise terms like ‘M phase of mitotic cell cycle’, ‘nuclear division’, ‘mitosis’, ‘cell division’, ‘microtubule-based process’, ‘microtubule cytoskeleton organization’, ‘regulation of microtubule-based process’, ‘mitotic prometaphase’ and ‘regulation of microtubule cytoskeleton organization’ (Figure 2A). With respect to cellular components (CC), GO terms including ‘microtubule organizing center’, ‘organelle inner membrane’, ‘spindle’, ‘microtubule’, ’chromosome centromeric region’, ’intermediate filament cytoskeleton’ and ’centrosome’ were significantly enriched (Figure 2B). Accordingly, the molecular functions (MF) ‘microtubule binding’ and ‘tubulin binding’ were enriched (Figure 2C). The enriched GO terms revealed here with respect to BP, CC and MF emphasize the inductive effect of blood serum and strongly correlate with the reported proliferative effect of human blood serum on hCSCs.

### 2.2. Inhibition of TGFβRI/II Enhances Proliferation While TGFβ Possesses a Dual Role in Regard to Proliferation and Senescence in hCSCs

**Figure 3:**
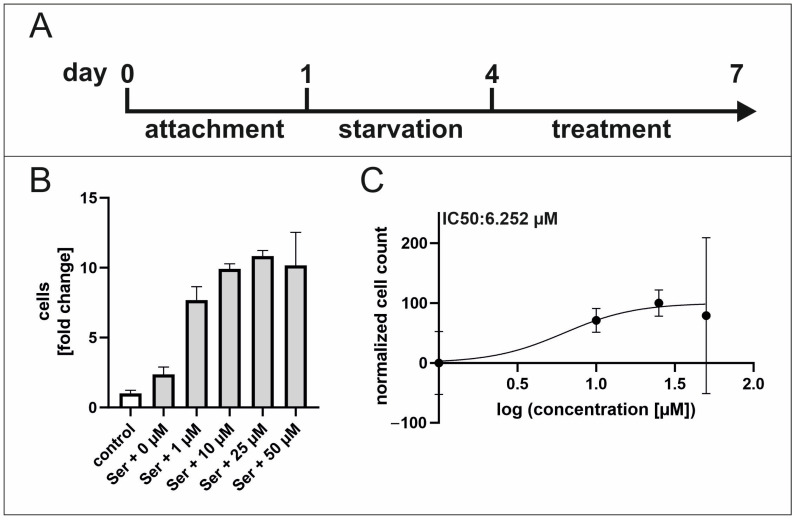
50 µM LY2109761 is effective for induction of proliferation in hCSCs. (**A**) After starvation, hCSCs were treated with human blood serum and TGFβR/II inhibitor. (**B**) Increasing concentrations of the selective TGFβRI/II inhibitor LY2109761 upon serum treatment indicate a proliferation enhancing effect in hCSCs with an effective concentration at 50 µM. (**C**) The IC50 value of the TGFβ RI/II inhibitor LY2109761 is 6.252 µM.

RNA-Seq revealed the differential gene expression of targets and inhibitors of the TGFβ1 pathway and the enrichment of proliferation-related GO terms after serum treatment in hCSCs. In addition, the downstream p38-MAPK signalling pathway was shown in previous studies to be significant for serum-mediated proliferation and migration of hCSCs [11,19], further underlining the potential role of TGFβ1 signalling in this regard. Consequently, the TGFβ1 signalling pathway was investigated to further clarify the molecular mechanisms of the beneficial effects of serum. Supplementation with increasing concentrations of TGFβ1 led to reduced proliferation of hCSCs compared to untreated cells, which indicates a suppressive role of TGFβ1 in this context. To gain deeper insights of the involvement of TGFβ in the observed suppressive effect on proliferation, the selective TGFβRI/II inhibitor LY2109761 [41] was applied upon serum treatment, leading to enhanced proliferation, further highlighting the central role of TGFβ signalling in this context. The proliferation assay showed increased proliferation of hCSCs correlating with increased concentration of inhibitor, with a maximum effect of a 6.2-fold induction of proliferation at 50 µM LY2109761 and an IC50 of 6.252 µM (Figure 3A–C).

**Figure 4:**
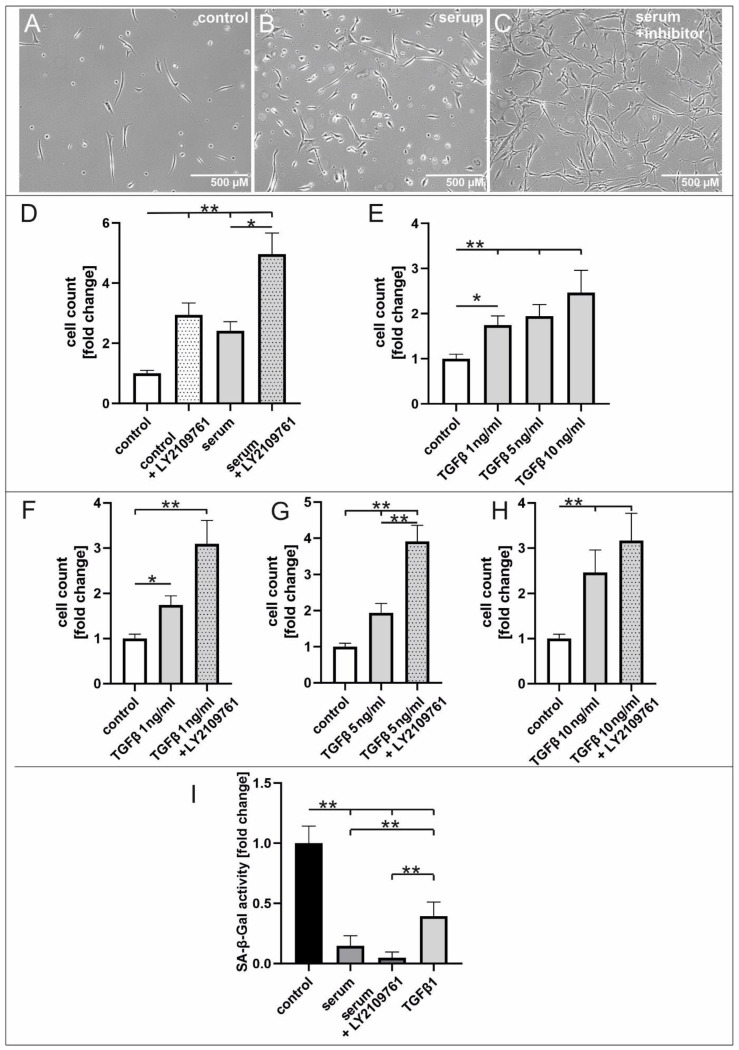
The dual role of TGFβ signalling in serum-mediated effects in hCSCs. (**A**–**C**) Upon serum treatment, the supplementation with LY2109761 leads to morphological changes of hCSCs. (**D**) The application of LY2109761 alone increases the proliferation higher than treatment with serum alone, while the proliferative effect of the serum is enhanced by LY210976. (**E**) TGFβ1 mediates proliferation in concentrations from 1 ng/mL to 10 ng/mL. (**F**–**H**) Simultaneous application of LY2109761 and TGFβ1 highly increases the cell proliferation of hCSCs regardless of the applied concentration of TGFβ1. (**I**) Serum reduces the SA-β-Gal activity in hCSCs, and this effect is further enhanced after addition of LY2109761. Application of TGFβ1 reduces senescence in hCSCs compared to untreated cells while it is significantly increased compared to serum-treated cells. (Mann–Whitney U two-tailed, ** *p* < 0.01; * *p* < 0.05).

In order to determine the role of TGFβ-signalling in serum-treated hCSCs, we performed proliferation and senescence assays using 50 µM of the TGFβRI/II inhibitor LY2109761. A strong and significant increase in hCSC proliferation was observed upon inhibition of TGFβRI/II and serum treatment. Notably, the application of the TGFβRI/II inhibitor LY2109761 also increased the proliferation of cells which were cultured in serum-deprived medium to a higher extent than in serum-treated cells (Figure 4D).

Besides the induction of proliferation, application of LY2109761resulted in morphological changes. Untreated hCSCs appeared as long spindle shaped cells with thin long processes and single granular cells. In contrast, serum-treated hCSCs were formed as single granular and spindle shaped cells with y-shaped cytoplasm. Supplementation with serum and LY2109761 also induced a spindle shaped morphology while here no single granular cells were visible. Particularly, a strong increase in cell–cell interactions were visible after TGFβRI/II inhibition as here each cell showed connections to other cells (Figure 4A–C). We investigated the amount of total TGFβ1 in several serum samples (Table 1) and found TGFβ1 levels ranging from 0.39 ng/mL to 1.10 ng/mL. Taken together, pooled serum induced proliferation of hCSCs in the same manner as application of 1 ng/mL TGFβ1 (see below).

We tested after inhibition with LY2109761, the effects of TGFβ1 on proliferation. They were investigated via supplementation of increasing TGFβ1 concentrations of 1 ng/mL, 5 ng/mL or 10 ng/mL, revealing a highly inductive effect. The application of low concentrations of TGFβ1 (1 ng/mL) could significantly increase proliferation whereas application of 10 ng/mL TGFβ1 had the same inductive effect as serum (Figure 4E). Strikingly, simultaneous application of the TGFβRI/II inhibitor and TGFβ1 highly increased the cell proliferation of hCSCs regardless of the applied concentration of TGFβ1 (Figure 4F–H), indicating a potential dual role of TGFβ signalling in hCSCs. Regarding senescence, serum significantly reduced the senescence-associated β-galactosidase (SA-β-Gal) activity in hCSCs, and this effect was further enhanced after addition of the inhibitor LY2109761. Application of TGFβ1 reduced senescence in hCSCs compared to untreated cells while the protective effect of TGFβ1 was smaller compared to serum-treated cells (Figure 4I). Here, a dual role of TGFβ1-signalling in regard to senescence in serum-treated hCSCs is likewise suggested.

### 2.3. Human Blood Serum Strongly Induces the Phosphorylation of SMAD2/3 in the Nucleus Which Is Significantly Reduced by the Application of the Selective Inhibitor LY2109761

**Figure 5:**
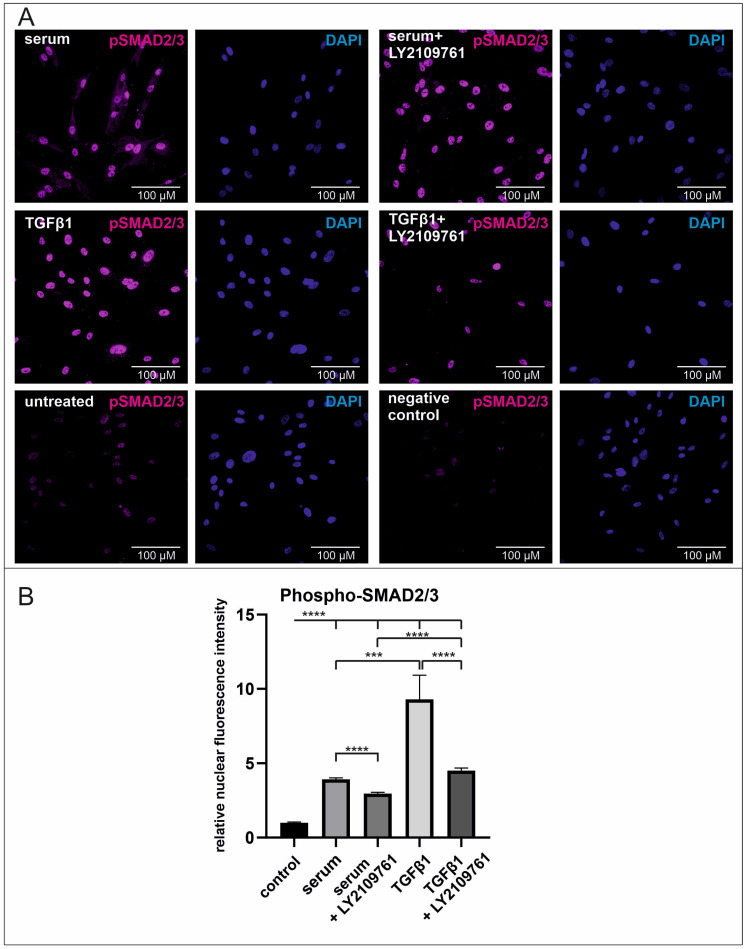
Phosphorylation of SMAD2/3 after serum treatment and inhibition of TGFβRI/II in hCSCs. (**A**) Immunocytochemical staining of hCSCs shows enriched amounts of pSMAD2/3 in the nucleus after treatment with serum or TGFβ1 which further increases after inhibition of TGFβRI/II, magnification ×63. (**B**) Quantification of the fluorescence signal reveals a significant decrease of nuclear pSMAD2/3 after application of TGFβRI/II inhibitor. (Kruskal–Wallis, *** *p* < 0.001; **** *p* < 0.0001).

To investigate the effect of human blood serum on TGFβ1 signalling and the function of LY2109761, phosphorylated SMAD2/3 (pSMAD2/3) was immunocytochemically determined in the nucleus of hCSCs. Serum treatment induced a three-fold increase of nuclear pSMAD2/3 compared to untreated hCSCs. Addition of the TGFβRI/II inhibitor LY2109761r significantly decreased this effect while the amount of nuclear phosphorylated SMAD2/3 was higher than in untreated cells. Supplementation with TGFβ1 resulted in an eight-fold increase of phosphorylated SMAD2/3 while this effect was reduced after the addition of LY2109761 (Figure 5A,B). These data underline the inductive effect of human blood serum on TGFβ1 signalling via SMAD2/3 phosphorylation and the successful reduction of this effect via the selective inhibitor LY2109761.

### 2.4. TGFβ RI Inhibition Reduces Serum-Mediated hCSC Proliferation

**Figure 6:**
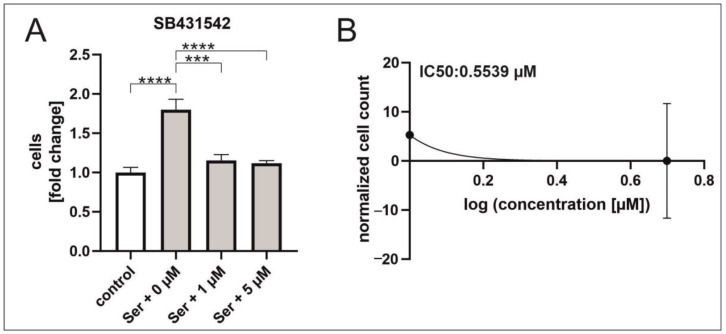
TGFβRI inhibition reduces serum-induced hCSC proliferation. (**A**) Application of TGFβRI inhibitor SB431542 reduces serum-mediated hCSC proliferation with an (**B**) IC_50_ of 0.5339 µM. (Ordinary one-way ANOVA, **** *p* < 0.0001; *** *p* < 0.001).

Following the striking observations of TGFβ1 and TGFβRI/II inhibition which both increased hCSC proliferation, a more detailed analysis was performed. The selective TGFβ type I receptor inhibitor, SB431542, was applied to serum-treated hCSCs. Contrary to the proliferative effect of TGFβRI/II inhibition, application of the TGFβRI inhibitor SB431542 resulted in a strong and significant reduction in the serum-mediated hCSC proliferation with an IC_50_ of 0.553 µM (Figure 6A,B).

### 2.5. Differential Global Gene Expression Indicates Upregulation of Proliferation-Related and Ras-Inactivating Genes after TGFβ RI/II Inhibition and Serum Treatment in hCSCs

**Figure 7:**
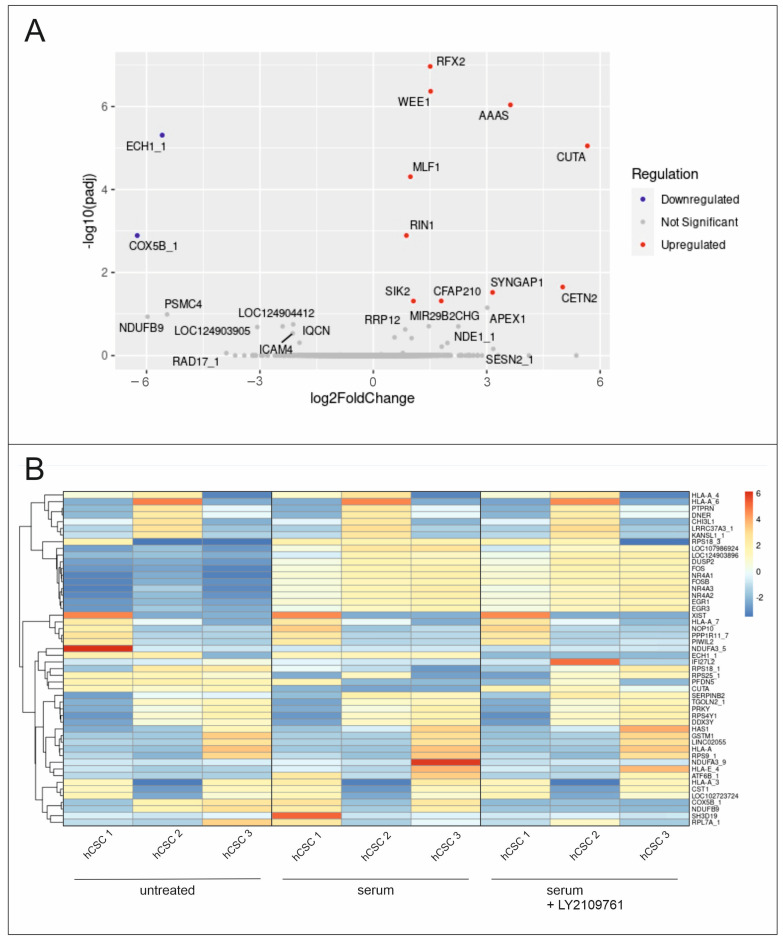
Gene expression analysis of serum- and LY2109761-treated hCSCs. (**A**) Volcano plot of differentially expressed genes in serum-treated hCSCs after TGFβ RI/II inhibition shows upregulation of ten genes and downregulation of two genes. Proliferation-related and Ras-inactivating genes are significantly upregulated after serum and inhibitor treatment in hCSCs. (**B**) The genes FOS, FOSB, NR4A and EGR are similarly upregulated in hCSCs treated with serum or serum with LY2109761 compared to untreated hCSCs.

To further clarify the underlying molecular basis of the observed effects, RNA-Seq was performed with serum- and LY2109761-treated hCSCs. The differential gene expression analysis revealed significant upregulation of proliferation-related genes, such as *WEE1* (*WEE1* Checkpoint Kinase), *CETN2* (Centrin2) and *MLF1* (Myeloid Leukemia Factor 1) in cells treated with serum and LY2109761 compared to serum treatment (Figure 7A). Additionally, Ras-inactivating genes such as *RIN1* (Ras and Rab interactor 1, Ras inhibitor 1) and *SYNGAP1* (Synaptic Ras GTPase Activating Protein 1) were differentially expressed (Figure 6A). Interestingly, the previously identified genes *FOS*, *FOSB*, *NR4A* and *EGR* were similarly upregulated in hCSCs treated with serum or serum with LY2109761 compared to untreated hCSCs (Figure 7B).

## 3. Discussion

The characteristics of adult human stem cells, such as the ability to self-renew and the high differentiation potential, make their presence crucial for the regeneration and homeostasis of the human organism [2]. Hence, the application of adult stem cells is a fast-growing field in regenerative medicine. This includes treatment of bone marrow failure, muscular dystrophy or neurogenerative diseases as well as the support of myocardial regeneration [6,7,8,42]. The exhaustion of stem cells and the increase of senescent cells both are hallmarks of ageing and contribute to the decline of the regenerative potential of the body [1,5]. Accordingly, the investigation of stem cell proliferation as a marker for stem cell fitness is necessary for developing new cell-based applications in regenerative medicine to counteract age-related diseases. Especially the human heart which was long considered as an organ with low regenerative capacity until self-renewing, clonogenic and multipotent cardiac stem cells were identified [6,11,12]. We previously isolated a Nestin^+^/S100^+^ cell population from human left atrial appendages which expresses the cardiac progenitor markers CD105, CD31 and Sca-1. This human cardiac stem cell population was able to form cardiospheres after clonal growth and differentiated into cardiomyocytes in vitro [11]. In terms of rejuvenation, several studies showed a beneficial effect of young blood, plasma or serum on old tissues and organs as well as on various stem cell populations [16,18]. We could successfully underline these effects by showing serum-mediated proliferation and senescence-reduction in hCSCs after application of human blood serum in previous studies [11,19].

Within this study, we showed that human blood serum induces enrichment of proliferation-related GO terms in hCSCs which is consistent with our previously observed serum-mediated proliferative effects [11]. Here, the upregulation of the biological processes associated with mitosis strongly indicate an immediate response to serum at a global gene expression level coherent to the observed proliferative phenotype. We previously identified the p38-MAPK/HSP27 axis as a partial mediator of serum-induced proliferation in hCSCs [19]. Consequently, making examinations of upstream signalling pathways is of great interest to further unravel the underlying molecular mechanisms of the serum-mediated proliferative effects in hCSCs. In this study we show the upregulation of 213 differentially expressed genes induced by serum. These comprise the TGFβ signalling target activating transcription factor 3 (ATF3) [39] and the endogenous TGFβ inhibitor nuclear receptor subfamily 4 group A member 1 (NR4A1) [40], which strongly implies the involvement of TGFβ-signalling in the serum-mediated response in hCSCs. ATF3, a known stress response gene belonging to the ATF/CREB family, has been reported to be induced by TGFβ-activated SMAD3 signalling in human epithelial cells [39]. Additionally, the transcription factor has not only been shown to be a target of TGFβ1 but also as a mediator of TGFβ signalling. In detail, it has previously been found that ATF3 induces proliferation and invasion and inhibits apoptosis via TGFβ1/SMAD2/3 signalling in keloid fibroblasts [43]. In contrast, the NR4A1 receptor has been shown to be an endogenous inhibitor of TGFβ signalling which mediates the repression of TGFβ target genes. The receptor therefore has been focused on in other studies to counter TGFβ-mediated signalling in several diseases [44,45]. The here observed involvement of both targets and inhibitors of the TGFβ pathway, makes further investigation to unravel the molecular mechanisms in hCSCs crucial to gain deeper insights on the serum-mediated effects in relation to proliferation. Especially focusing on cardiac development, the TGFβ signalling pathway has already been reported to be involved by several studies. Interestingly, the cardiac progenitor marker endoglin (CD105) is also known to be an ancillary TGFβ receptor and has been shown to be required for extraembryonic angiogenesis and heart development [46]. In several species, bone morphogenic proteins (BMPs), members of the TGFβ superfamily, have also been found to mediate cardiac development. This comprises the participation of BMPs and SMADs during heart development in terms of endocardial cushion formation, septation and specification of the left/right axis [30,31,32,33,34,35]. Further, the TGFβ signalling pathway has been reported to be crucial during vascular development [36]. Additionally, previous studies showed the secretion of TGFβ1 by cardiac fibroblasts and cardiomyocytes resulting in the induction of myofibroblast transdifferentiation [37,38,47]. Based on the here-demonstrated induction of TGFβ-related genes after serum treatment in hCSCs and the reported involvement of TGFβ signalling in several cardiac processes, we analysed proliferation. We analysed six different samples of human serum with a TGFβ1-specific ELISA and detected amounts of about 1 ng/mL TGFβ1, which is in accordance with the current literature [48]. In line with these concentrations, we observed a similar induction of proliferation (2-fold) with either pooled sera or recombinant TGFß1 (1 ng/mL). A selective TGFβRI/II inhibitor LY2109761 which negatively affects the phosphorylation of SMAD2/3 was utilized. The serum-mediated proliferative effects of human blood serum on hCSCs were significantly increased by LY2109761. A strong proliferation inducing effect of TGFβ1 on hCSCs was measured. Strikingly, within this study, the application of TGFβRI/II inhibitor significantly increased the proliferation of serum-deprived cultivated hCSCs whereby the effect was higher than in serum-treated cells. Coherently, a strong and significant increase in hCSC proliferation was also observed after serum treatment and inhibition of the TGFβRI/II. As the application of different concentrations of TGFβ1 and simultaneous application of the TGFβRI/II inhibitor LY2109761 increased proliferation, while application of TGFβ1 alone also induced proliferation, a dual role of TGFβ-signalling in hCSCs is strongly suggested. Furthermore, human blood serum significantly reduced senescence as measured by SA-β-Gal activity, while supplementation with LY2109761 further increased this beneficial serum effect. Application of TGFβ likewise reduced senescence in hCSCs; however, this effect was to a lesser extent than in serum or serum- and LY210971-treated hCSCs. Our results again indicate a dual role of TGFβ1 signalling regarding senescence in hCSCs and a prevention of serum-induced hyperproliferation in hCSCs via TGFβ1 signalling. In this context it has been previously reported that the loss of TGFβ1 expression resulted in hyperproliferation of skin and skin tumours of mice [49]. Further, a senescence-inducing effect of TGFβ has been shown in human diploid fibroblasts, bronchial epithelial cells and cancer cells [25,50,51,52]. Additionally, an inductive effect of human blood serum on TGFβ1 signalling via SMAD2/3 phosphorylation and the successful reduction of this effect via LY2109761 was demonstrated by analysing phosphorylation of nuclear SMAD2/3 in hCSCs. The observed higher inductive effect of TGFβ1 compared to the effect of the serum may be due to lower amounts of TGFβ1 in the serum or TGFβ inhibitors present in the serum.

To extend our understanding of the proliferative effects of TGFβRI/II inhibition, the selective TGFβRI inhibitor SB431542 was applied, and it significantly reduced proliferation in serum-treated hCSCs. This proliferation-reducing effect after selective inhibition of TGFβRI has also been seen in human tenon’s fibroblast [53]. Moreover, prevention of SMAD2/3 phosphorylation via TGFβRI inhibition potentially induced the upregulation of the TGFβ-non-SMAD pathway, which comprises the activation of Ras and Rho signalling, which have been strongly linked to cell proliferation [11,54,55,56,57,58]. While LY2109761 inhibits the kinase domains of both, TGFβ receptor type I and II [41], SB431542 selectively inhibits ALK5 TGFβ receptor type I [59]. We therefore conclude that the proliferative effect of LY2109761 could be a result of TGFβ receptor type II inhibition. This hypothesis could be strengthened by the fact that TGFβ receptor type II is constitutively active by autophosphorylation and this autophosphorylation is blocked by LY2109761 [41]. In turn, we suggest that TGFβRII potentially prevents hyperproliferation by activating pathways or signal cascades negatively regulating proliferation. In this regard, an increased TGFβRII expression has already been shown to directly correlate with a higher CDKN1A/p21 expression which is associated with senescence induction [60,61]. Further, the mutational inactivation of TGFβRII has been discussed to increase G1/S transition kinase activity of CDK4 leading to colon cancer cell proliferation [62]. Additionally, the type II receptor has been shown to act as a G-protein-coupled receptor kinase (GRK), thereby showing that activity of the receptor alone can independently induce other signalling pathways [63]. Besides, previous studies have been demonstrating a direct regulatory function of TGFβRII on non-canonical Rho-associated protein kinase (Rho-ROCK) pathway [64]. Interestingly, likewise to our observations after LY2109761 treatment of hCSCs, the inhibition of ROCK via Y-27632 has been shown to promote cell viability and proliferation of ESCs, ESC-derived endothelial cells and primary keratinocytes while it also has been shown to reduce senescence [65]. Notably, ROCK inhibition by utilizing Y-27632 has been previously reported to reduce Ras-induced senescence in skin keratinocytes [66]. Within our study, RNA-Seq revealed the upregulation of Ras-inactivating genes after serum and TGFβRI/II inhibition via LY2109761 which strongly indicates the involvement of the ROCK-signalling pathway in this context. Based on these results, we conclude that TGFβRII potentially regulates Ras-mediated prevention of hyperproliferation in hCSCs.

We used RNA-Seq to investigate the effects of TGFβRI/II inhibition in contrast to the proliferation reducing effects of TGFβRI inhibition in hCSCs. Interestingly, in accordance with our observed proliferative effects of TGFβ RI/II inhibition upon serum treatment, proliferation related genes were significantly upregulated. These comprise *WEE1* (*WEE1 Checkpoint Kinase*), *CETN2* (*Centrin2*) and *MLF1* (*Myeloid Leukemia Factor 1*). In previous studies we showed that human blood serum induces proliferation and Ras signalling in hCSCs via Gene Ontology (GO) Term Analysis [11]. The Ras signalling pathway is known to be involved in proliferation, differentiation, morphology and apoptosis [67,68]. A direct target of Ras signalling is the induction of the cell cycle via expression of D cyclins which subsequently activate Cdk4 and Cdk6 through phosphorylation allowing them to inactive the pocket proteins resulting in cell cycle progression from G1 to S phase [57,69,70,71]. The activation of Ras signalling, therefore, has been shown to lead to proliferation and migration in several cell types, such as mouse embryonic fibroblasts [72]. Ras has also been reported to act as a proto-oncogene with mutations of the Ras genes highly occurring in pancreatic, lung and colorectal carcinomas [67,73,74]. On the other hand, Ras has been shown to play a role in senescence in primary human or rodent cells, resulting in a permanent G1 arrest [75]. Here, differential gene expression analysis revealed a significant upregulation of Ras-inactivating genes such as *RIN1*, and *SYNGAP1* after serum treatment and TGFβRI/II inhibition. RIN1 (Ras and Rab interactor 1, Ras inhibitor 1) is a known Ras inhibitor while SYNGAP1 (Synaptic Ras GTPase Activating Protein 1) is known to negatively regulate Ras [76,77]. Best to our knowledge the presence of SYNGAP1 has not been reported in the context of cardiac regeneration so far. Considering the here observed selective effects of TGFβRI/II inhibition in the context of proliferation and senescence in hCSCs, it is suggested that TGFβRII-mediated regulation of non-SMAD signalling prevents serum-mediated hyperproliferation of hCSCs via TGFβRII-mediated Ras-induced senescence. Further comparison of the differential gene expression of hCSCs treated with either serum or serum with LY2109761 and untreated hCSCs, revealed the upregulation of the previously identified genes FOS, FOSB, NR4A and EGR as mediators of hCSC proliferation. In accordance, the FOS protein family has been reported to modulate proliferation in several cell types, such as osteosarcoma cells, osteoblasts and human lung cancer cell lines [78,79,80,81]. Further, the NR4A nuclear receptor family is known to induce endothelial cell proliferation, smooth muscle cells and cancer cells [82,83,84,85,86]. Likewise, the transcription factor EGR1 has been demonstrated to modulate the proliferation of astrocytes, smooth muscle cells and gastric cancer cells [87,88,89,90]. Overall, our results underline the upregulation of these genes in the context of serum-induced proliferation of hCSCs.

In conclusion, we demonstrate proliferation-related responses of hCSCs to serum treatment at a gene expression level which were revealed by Gene Ontology while involvement of the TGFβ signalling pathway was indicated. Subsequent inhibition of the TGFβRI/II and simultaneous serum treatment significantly increased proliferation and reduced senescence. Both, human blood serum and TGFβ1, strongly induced the phosphorylation of nuclear SMAD2/3. Altogether, a dual role of TGFβ1 signalling in hCSCs is suggested. Contrary to inhibition of TGFβRI and II, inhibition of TGFβRI resulted in reduced proliferation after serum treatment. RNA-Seq revealed the significant upregulation of proliferation-related and Ras-inactivating genes of hCSCs treated with serum and TGFβRI/II. As observed here, the induction of proliferation is potentially based on the TGFβRII inhibition; we conclude that TGFβRII regulates (non-canonical) pathways which mediate the prevention of hyperproliferation in hCSCs. In detail, we suggest that the observed proliferation is mediated by repression of Ras signalling. This dual role of TGFβ may help to explain the characteristics of molecular regulation in tissues with low turnover such as the heart and lead to new therapeutic approaches in the future.

## 4. Materials and Methods

### 4.1. Isolation and Cultivation of Human Cardiac Stem Cells

Adult human cardiac cells (hCSCs) have been isolated and cultivated via explant cultures from human atrial left appendages which have been routinely removed during surgery in accordance with local and international guidelines (declaration of Helsinki). Ethical approval for isolation and experimental procedures was conducted by the ethics commission of the Ruhr-University Bochum (Faculty of Medicine, located in Bad Oeynhausen, with the approval number eP-2016-148). Isolation and cultivation were performed as described previously [11].

### 4.2. Proliferation Assay

For the proliferation assays, hCSCs were pre-cultivated in TC25 flasks (Sarstedt AG and Co., Nürmbrecht, Germany). After detachment using Trypsin-EDTA (Sigma Aldrich, St Louis, MO, USA), 1 × 10^3^ cells per well were seeded in a 0.1% gelatin-type-B (Sigma Aldrich, Taufkirchen, Germany)-coated 96-TC plate (Sarstedt AG and Co., Nürmbrecht, Germany) in hCSC medium which consisted of DMEM-F12 (Life Technologies, Darmstadt, Germany), 10% foetal calf serum, L-glutamine (2 mmol/L), bFGF (5 ng/mL) (Miltenyi Biotech, Bergisch Gladbach, Germany), EGF (10 ng/mL) (Preprotech, Hamburg, Germany) and Penicillin/Streptomycin (10 mg/mL) (Sigma Aldrich, Taufkirchen, Germany). Triplicates were performed for each condition. After 24 h, the medium was changed to the starvation medium which contained DMEM-F12 (Life Technologies, Darmstadt, Germany), L-glutamine (2 mmol/L), bFGF (5 ng/mL) (Miltenyi Biotech, Bergisch Gladbach, Germany), EGF (10 ng/mL) (Preprotech, Hamburg, Germany) and Penicillin/Streptomycin (10 mg/mL) (Sigma Aldrich, Taufkirchen, Germany) for 72 h. To calculate the IC50 value, 10% pooled blood serum and concentrations from 0 to 50 µM of selective TGFβRI/II inhibitor LY2109761 (Selleckchem) or 0 to 10 µM TGFβRI inhibitor SB431542 (abcam, Cambridge, UK) dissolved in DMSO (Sigma Aldrich, Taufkirchen, Germany) was applied. The amounts of DMSO in each well were equal to the maximum concentration of DMSO, respectively. For determination of the role of TGFβRI/II in the serum-mediated proliferative effects on hCSCs human blood serum, 50 µM LY2109761 was applied. After 72 h at 37 °C under hypoxic conditions with 5% CO_2_ and 5% O_2_ at 37 °C, 10 µL Orangu Cell Counting Solution (Cell Guidance Systems, Cambridge, UK) was added to each well for 2 h at 37 °C. Absorbance was measured at 450 nm and the cell count was calculated by the formula derived from a previously determined calibration straight line. For calibration, hCSCs from the same suspension were seeded in a 0.1% gelatin-type-B-coated TC-96 plate with 0, 250, 500, 750, 1000, 1500, 2000, 2500 and 3000 cells per well. After allowing the cells to attach to the surface for 1.5 h, 10 µL Orangu Cell Counting Solution (Cell Guidance Systems, Cambridge, UK) was added for 2 h and the absorbance was measured at 450 nm. Clinically accredited therapeutic human blood plasma samples were collected from six healthy young (age < 20 years) male donors via a routine blood donation service (Institute for Laboratory- and Transfusionmedicine, Bad Oenhausen, Germany). Serum was isolated as described previously [11]. Levels of TGFβ1 were quantified using an ELISA Kit (88-8350-22, Invitrogen, Thermo Fisher, Waltham, MA, USA) according to the manufacturer’s guidelines. Values within the linear range of the serum dilution series were used for calculation of the TGFβ1 amount.

### 4.3. Senescence-Associated Quantitative β-Galactosidase Assay

A total of 1 × 10^5^ human cardiac stem cells were seeded in 0.1% gelatin-type-B-coated TC100 dishes. Cultivation and treatment were equal to the conditions described for the proliferation assay. Triplicates were performed. After washing six times with PBS, 300 µL 1× lysis buffer containing 5 mM CHAPS, 40 mM citric acid, 40 mM sodium phosphate, 0.5 mM benzamidine and 0.25 mM PMSF was added. The cells were detached by scraping and transferred to a 1.5 mL reaction tube and froze at −80 °C. Subsequent to thawing, the samples were centrifuged at 12,000× *g* for 5 min and 100 µL of the lysate was mixed with 100 µL 2× reaction buffer containing 40 mM citric acid, 40 mM sodium phosphate, 300 mM NaCl, 300 mM β-mercaptoethanol, 4 mM MgCl_2_ and 1.7 mM MUG. After incubation at 37 °C for one hour, 50 µL of the reaction mix was added to 150 µL 400 mM sodium carbonate stop solution. Then, 150 µL was transferred to a black 96-well plate and quantification was performed via a TECAN reader with excitation 360 nm, emission 465 nm and integration 40 µs. The senescence-associated β-galactosidase activity was normalized to the amount of the total isolated protein.

### 4.4. RNA Sequencing and Analysis

In total, 5 × 10^5^ hCSCs from three male donors were seeded in 0.1% gelatin-type-B (Sigma Aldrich, Taufkirchen, Germany)-coated TC100 dishes (Sarstedt AG and Co., Nürmbrecht, Germany) in hCSC medium (described previously) and cultivated for 24 h. Subsequently, the medium was changed to the starvation medium (described previously) for a further 24 h. An amount of 50 µM of LY2109761 or the equivalent amount of DMSO was then applied for one hour for pre-treatment, followed by one-hour treatment with either 10% pooled human blood serum from six young males or 10% pooled human blood serum from six young males and 50 µM LY2109761. RNA was isolated using the NucleoSpin RNA Kit (Macherey-Nagel GmbH & Co. KG, Düren, Germany) according to the manufacturer’s guidelines. For determination of the amount of isolated RNA, a nanodrop (Peqlab, Erlangen, Germany) was used. The samples were stored at −80 °C. mRNA library preparation and sequencing were conducted by Novogene (Cambridge, UK) with NovaSeq 6000 30 M reads PE150. Using HISAT2 (version 2.1.0-4), the reads were aligned to the reference genome GRCh38.p14. Feature Counts (version 2.0.0) was used to quantify the read number after mapping. For differential gene expression analysis, the DESeq2 R package was used. Gene Ontology (GO) term enrichment and the Kyoto Encyclopedia of Genes and Genomes (KEGG) pathway analysis was performed by using the gage package in R (version1.3.1093). For KEGG analysis, the Pathview R package and for GO-Term analysis, the org.Hs.eg.db R package provided databases.

### 4.5. Immunocytochemical Staining

For immunocytochemical staining, cells were cultivated in 8-well slides (ibidi, cells in focus, Gräfelfing, Germany) as described for the RNA sequencing analysis. The medium was discarded, and the cells were washed one time with PBS followed by fixation at room temperature with 4% PFA for 15 min. After washing three times with PBS, the cells were permeabilized and blocked with 0.02% PBT containing 5% goat serum for 30 min. Subsequently, cells were washed one time with PBS. The primary antibody phosphoSMAD2/3 (Carlsbad, CA, USA) was applied overnight at 4 °C, diluted 1:300, in blocking solution. The second antibody goat anti rabbit alexa 555 (Life Technologies, Thermo Fisher Scientific, Waltham, MA, USA) was diluted 1:300 in blocking solution and applied for one hour in the dark at room temperature. After washing three times with PBS, nuclei were stained via 49,6-diamino-2-phenylindole (DAPI, 1 µg/mL) (Sigma-Aldrich, Taufkirchen, Germany) for 15 min, followed by two washing steps with PBS and one time with H_2_O. Mowiol-4-88 was mounted to cover the cells. Fluorescence imaging was performed by confocal laser scanning microscopy (LSM780; Carl Zeiss; Jena, Germany). For analysis, ZEN software (version 2011 SP7) and ImageJ (https://imagej.net/ij/, accessed on 8 January 2024) were used.

## 5. Patents

Parts of this research article were filed for a patent application entitled “Method and medium for cultivating animal cells”.

## Figures and Tables

**Table 1:** TGFβ1 levels in the human blood serum samples. Quantification of the TGFβ1 levels in the serum range from 0.39 ng/mL to 1.10 ng/mL.

Sample	TGFβ1 [ng/mL]
Serum 1	0.39
Serum 2	0.84
Serum 3	1.10
Serum 4	1.17
Serum 5	0.93
Serum 6	1.10

## Data Availability

Data is contained within the article.

## References

[1] López-Otín C., Blasco M.A., Partridge L., Serrano M., Kroemer G. (2013). The Hallmarks of Aging. Cell.

[2] He S., Nakada D., Morrison S.J. (2009). Mechanisms of Stem Cell Self-Renewal. Annu. Rev. Cell Dev. Biol..

[3] Wagers A.J., Weissman I.L. (2004). Plasticity of Adult Stem Cells. Cell.

[4] Cooper G.M., Cooper G.M. (2000). The Cell.

[5] Honoki K. (2017). Preventing Aging with Stem Cell Rejuvenation: Feasible or Infeasible?. World J. Stem Cells.

[6] Beltrami A.P., Barlucchi L., Torella D., Baker M., Limana F., Chimenti S., Kasahara H., Rota M., Musso E., Urbanek K. (2003). Adult Cardiac Stem Cells Are Multipotent and Support Myocardial Regeneration. Cell.

[7] Meza-Zepeda L.A., Noer A., Dahl J.A., Micci F., Myklebost O., Collas P. (2008). High-Resolution Analysis of Genetic Stability of Human Adipose Tissue Stem Cells Cultured to Senescence. J. Cell. Mol. Med..

[8] Mezey E., Key S., Vogelsang G., Szalayova I., Lange G.D., Crain B. (2003). Transplanted Bone Marrow Generates New Neurons in Human Brains. Proc. Natl. Acad. Sci. USA.

[9] Laflamme M.A., Murry C.E. (2011). Heart Regeneration. Nature.

[10] Aguilar-Sanchez C., Michael M., Pennings S. (2018). Cardiac Stem Cells in the Postnatal Heart: Lessons from Development. Stem Cells Int..

[11] Höving A.L., Schmidt K.E., Merten M., Hamidi J., Rott A.-K., Faust I., Greiner J.F.W., Gummert J., Kaltschmidt B., Kaltschmidt C. (2020). Blood Serum Stimulates P38-Mediated Proliferation and Changes in Global Gene Expression of Adult Human Cardiac Stem Cells. Cells.

[12] Bearzi C., Rota M., Hosoda T., Tillmanns J., Nascimbene A., De Angelis A., Yasuzawa-Amano S., Trofimova I., Siggins R.W., LeCapitaine N. (2007). Human Cardiac Stem Cells. Proc. Natl. Acad. Sci. USA.

[13] Smits A.M., van Vliet P., Metz C.H., Korfage T., Sluijter J.P., Doevendans P.A., Goumans M.-J. (2009). Human Cardiomyocyte Progenitor Cells Differentiate into Functional Mature Cardiomyocytes: An in Vitro Model for Studying Human Cardiac Physiology and Pathophysiology. Nat. Protoc..

[14] Fuentes T., Kearns-Jonker M. (2013). Endogenous Cardiac Stem Cells for the Treatment of Heart Failure. Stem Cells Cloning.

[15] Villeda S.A., Plambeck K.E., Middeldorp J., Castellano J.M., Mosher K.I., Luo J., Smith L.K., Bieri G., Lin K., Berdnik D. (2014). Young Blood Reverses Age-Related Impairments in Cognitive Function and Synaptic Plasticity in Mice. Nat. Med..

[16] Conboy I.M., Conboy M.J., Wagers A.J., Girma E.R., Weissman I.L., Rando T.A. (2005). Rejuvenation of Aged Progenitor Cells by Exposure to a Young Systemic Environment. Nature.

[17] Yousefzadeh M.J., Schafer M.J., Hooten N.N., Atkinson E.J., Evans M.K., Baker D.J., Quarles E.K., Robbins P.D., Ladiges W.C., LeBrasseur N.K. (2018). Circulating Levels of Monocyte Chemoattractant Protein-1 as a Potential Measure of Biological Age in Mice and Frailty in Humans. Aging Cell.

[18] Loffredo F.S., Steinhauser M.L., Jay S.M., Gannon J., Pancoast J.R., Yalamanchi P., Sinha M., Dall’Osso C., Khong D., Shadrach J.L. (2013). Growth Differentiation Factor 11 Is a Circulating Factor That Reverses Age-Related Cardiac Hypertrophy. Cell.

[19] Höving A.L., Schmitz J., Schmidt K.E., Greiner J.F.W., Knabbe C., Kaltschmidt B., Grünberger A., Kaltschmidt C. (2021). Human Blood Serum Induces P38-MAPK- and Hsp27-Dependent Migration Dynamics of Adult Human Cardiac Stem Cells: Single-Cell Analysis via a Microfluidic-Based Cultivation Platform. Biology.

[20] Roberts A.B., Wakefield L.M. (2003). The Two Faces of Transforming Growth Factor β in Carcinogenesis. Proc. Natl. Acad. Sci. USA.

[21] Seoane J. (2008). The TGFß Pathway as a Therapeutic Target in Cancer. Clin. Transl. Oncol..

[22] Moustakas A., Pardali K., Gaal A., Heldin C.-H. (2002). Mechanisms of TGF-β Signaling in Regulation of Cell Growth and Differentiation. Immunol. Lett..

[23] Zhang Y., Alexander P.B., Wang X.-F. (2017). TGF-β Family Signaling in the Control of Cell Proliferation and Survival. Cold Spring Harb. Perspect. Biol..

[24] Massagué J. (2012). TGFβ Signalling in Context. Nat. Rev. Mol. Cell Biol..

[25] Tominaga K., Suzuki H.I. (2019). TGF-β Signaling in Cellular Senescence and Aging-Related Pathology. Int. J. Mol. Sci..

[26] Tzavlaki K., Moustakas A. (2020). TGF-β Signaling. Biomolecules.

[27] Massagué J., Sheppard D. (2023). TGF-β Signaling in Health and Disease. Cell.

[28] Grainger D.J., Mosedale D.E., Metcalfe J.C., Weissberg P.L., Kemp P.R. (1995). Active and Acid-Activatable TGF-β in Human Sera, Platelets and Plasma. Clin. Chim. Acta.

[29] Shi M., Zhu J., Wang R., Chen X., Mi L., Walz T., Springer T.A. (2011). Latent TGF-β Structure and Activation. Nature.

[30] Gaussin V., Van de Putte T., Mishina Y., Hanks M.C., Zwijsen A., Huylebroeck D., Behringer R.R., Schneider M.D. (2002). Endocardial Cushion and Myocardial Defects after Cardiac Myocyte-Specific Conditional Deletion of the Bone Morphogenetic Protein Receptor ALK3. Proc. Natl. Acad. Sci. USA.

[31] Kennedy K.A., Porter T., Mehta V., Ryan S.D., Price F., Peshdary V., Karamboulas C., Savage J., Drysdale T.A., Li S.-C. (2009). Retinoic Acid Enhances Skeletal Muscle Progenitor Formation and Bypasses Inhibition by Bone Morphogenetic Protein 4 but Not Dominant Negative β-Catenin. BMC Biol..

[32] Furtado M.B., Solloway M.J., Jones V.J., Costa M.W., Biben C., Wolstein O., Preis J.I., Sparrow D.B., Saga Y., Dunwoodie S.L. (2008). BMP/SMAD1 Signaling Sets a Threshold for the Left/Right Pathway in Lateral Plate Mesoderm and Limits Availability of SMAD4. Genes Dev..

[33] Chen J.-N., van Eeden F.J.M., Warren K.S., Chin A., Nüsslein-Volhard C., Haffter P., Fishman M.C. (1997). Left-Right Pattern of Cardiac BMP4 May Drive Asymmetry of the Heart in Zebrafish. Development.

[34] Camenisch T.D., Molin D.G.M., Person A., Runyan R.B., Gittenberger-de Groot A.C., McDonald J.A., Klewer S.E. (2002). Temporal and Distinct TGFβ Ligand Requirements during Mouse and Avian Endocardial Cushion Morphogenesis. Dev. Biol..

[35] Kim R.Y., Robertson E.J., Solloway M.J. (2001). Bmp6 and Bmp7 Are Required for Cushion Formation and Septation in the Developing Mouse Heart. Dev. Biol..

[36] Folkman J., D’Amore P.A. (1996). Blood Vessel Formation: What Is Its Molecular Basis?. Cell.

[37] Pappritz K., Savvatis K., Koschel A., Miteva K., Tschöpe C., Van Linthout S. (2018). Cardiac (Myo)Fibroblasts Modulate the Migration of Monocyte Subsets. Sci. Rep..

[38] van Nieuwenhoven F.A., Turner N.A. (2013). The Role of Cardiac Fibroblasts in the Transition from Inflammation to Fibrosis Following Myocardial Infarction. Vasc. Pharmacol..

[39] Kang Y., Chen C.-R., Massagué J. (2003). A Self-Enabling TGFβ Response Coupled to Stress Signaling: Smad Engages Stress Response Factor ATF3 for Id1 Repression in Epithelial Cells. Mol. Cell.

[40] Palumbo-Zerr K., Zerr P., Distler A., Fliehr J., Mancuso R., Huang J., Mielenz D., Tomcik M., Fürnrohr B.G., Scholtysek C. (2015). Orphan Nuclear Receptor NR4A1 Regulates Transforming Growth Factor-β Signaling and Fibrosis. Nat. Med..

[41] Melisi D., Ishiyama S., Sclabas G.M., Fleming J.B., Xia Q., Tortora G., Abbruzzese J.L., Chiao P.J. (2008). LY2109761, a Novel Transforming Growth Factor Beta Receptor Type I and Type II Dual Inhibitor, as a Therapeutic Approach to Suppressing Pancreatic Cancer Metastasis. Mol. Cancer Ther..

[42] Toma C., Pittenger M.F., Cahill K.S., Byrne B.J., Kessler P.D. (2002). Human Mesenchymal Stem Cells Differentiate to a Cardiomyocyte Phenotype in the Adult Murine Heart. Circulation.

[43] Wang X.-M., Liu X.-M., Wang Y., Chen Z.-Y. (2021). Activating Transcription Factor 3 (ATF3) Regulates Cell Growth, Apoptosis, Invasion and Collagen Synthesis in Keloid Fibroblast through Transforming Growth Factor Beta (TGF-Beta)/SMAD Signaling Pathway. Bioengineered.

[44] Zeng X., Yue Z., Gao Y., Jiang G., Zeng F., Shao Y., Huang J., Yin M., Li Y. (2018). NR4A1 Is Involved in Fibrogenesis in Ovarian Endometriosis. Cell. Physiol. Biochem..

[45] Mihara K., Saifeddine M., Hollenberg M.D. (2022). Metformin Down-Regulates TGF Beta Signal Transduction and Production of PAR2 N-Terminus Cleaving Protease Activity in an NR4a1 Dependent Manner in a PC3 Prostate Cancer Cell Line. FASEB J..

[46] Arthur H.M., Ure J., Smith A.J.H., Renforth G., Wilson D.I., Torsney E., Charlton R., Parums D.V., Jowett T., Marchuk D.A. (2000). Endoglin, an Ancillary TGFβ Receptor, Is Required for Extraembryonic Angiogenesis and Plays a Key Role in Heart Development. Dev. Biol..

[47] Spillmann F., Miteva K., Pieske B., Tschöpe C., Van Linthout S. (2015). High-Density Lipoproteins Reduce Endothelial-to-Mesenchymal Transition. Arterioscler. Thromb. Vasc. Biol..

[48] Grainger D.J., Mosedale D.E., Metcalfe J.C. (2000). TGF-β in Blood: A Complex Problem. Cytokine Growth Factor Rev..

[49] Glick A.B., Kulkarni A.B., Tennenbaum T., Hennings H., Flanders K.C., O’Reilly M., Sporn M.B., Karlsson S., Yuspa S.H. (1993). Loss of Expression of Transforming Growth Factor Beta in Skin and Skin Tumors Is Associated with Hyperproliferation and a High Risk for Malignant Conversion. Proc. Natl. Acad. Sci. USA.

[50] Frippiat C., Chen Q.M., Zdanov S., Magalhaes J.P., Remacle J., Toussaint O. (2001). Subcytotoxic H2O2 Stress Triggers a Release of Transforming Growth Factor-Beta 1, Which Induces Biomarkers of Cellular Senescence of Human Diploid Fibroblasts. J. Biol. Chem..

[51] Minagawa S., Araya J., Numata T., Nojiri S., Hara H., Yumino Y., Kawaishi M., Odaka M., Morikawa T., Nishimura S.L. (2011). Accelerated Epithelial Cell Senescence in IPF and the Inhibitory Role of SIRT6 in TGF-β-Induced Senescence of Human Bronchial Epithelial Cells. Am. J. Physiol. Lung Cell Mol. Physiol..

[52] Matsuda S., Revandkar A., Dubash T.D., Ravi A., Wittner B.S., Lin M., Morris R., Burr R., Guo H., Seeger K. (2023). TGF-β in the Microenvironment Induces a Physiologically Occurring Immune-Suppressive Senescent State. Cell Rep..

[53] Nassar K., Grisanti S., Tura A., Lüke J., Lüke M., Soliman M., Grisanti S. (2014). A TGF-β Receptor 1 Inhibitor for Prevention of Proliferative Vitreoretinopathy. Exp. Eye Res..

[54] Mu Y., Gudey S.K., Landström M. (2012). Non-Smad Signaling Pathways. Cell Tissue Res..

[55] Zhang Y.E. (2009). Non-Smad Pathways in TGF-β Signaling. Cell Res..

[56] Tu S., Huang W., Huang C., Luo Z., Yan X. (2019). Contextual Regulation of TGF-β Signaling in Liver Cancer. Cells.

[57] Coleman M.L., Marshall C.J., Olson M.F. (2004). RAS and RHO GTPases in G1-Phase Cell-Cycle Regulation. Nat. Rev. Mol. Cell Biol..

[58] Provenzano P.P., Keely P.J. (2011). Mechanical Signaling through the Cytoskeleton Regulates Cell Proliferation by Coordinated Focal Adhesion and Rho GTPase Signaling. J. Cell Sci..

[59] Inman G.J., Nicolás F.J., Callahan J.F., Harling J.D., Gaster L.M., Reith A.D., Laping N.J., Hill C.S. (2002). SB-431542 Is a Potent and Specific Inhibitor of Transforming Growth Factor-Beta Superfamily Type I Activin Receptor-like Kinase (ALK) Receptors ALK4, ALK5, and ALK7. Mol. Pharmacol..

[60] Rojas A., Padidam M., Cress D., Grady W.M. (2009). TGF-β Receptor Levels Regulate the Specificity of Signaling Pathway Activation and Biological Effects of TGF-β. Biochim. Biophys. Acta.

[61] Wagner K.-D., Wagner N. (2022). The Senescence Markers p16INK4A, p14ARF/p19ARF, and P21 in Organ Development and Homeostasis. Cells.

[62] Grady W.M., Willis J.E., Trobridge P., Romero-Gallo J., Munoz N., Olechnowicz J., Ferguson K., Gautam S., Markowitz S.D. (2006). Proliferation and Cdk4 Expression in Microsatellite Unstable Colon Cancers with TGFBR2 Mutations. Int. J. Cancer.

[63] Qiu T., Wu X., Zhang F., Clemens T.L., Wan M., Cao X. (2010). TGF-Beta Type II Receptor Phosphorylates PTH Receptor to Integrate Bone Remodelling Signalling. Nat. Cell Biol..

[64] Vardouli L., Vasilaki E., Papadimitriou E., Kardassis D., Stournaras C. (2008). A Novel Mechanism of TGFbeta-Induced Actin Reorganization Mediated by Smad Proteins and Rho GTPases. FEBS J..

[65] Svensmark J.H., Brakebusch C. (2019). Rho GTPases in Cancer: Friend or Foe?. Oncogene.

[66] Lee A.J., Fraser E., Flowers B., Kim J., Wong K., Cataisson C., Liu H., Yang H., Lee M.P., Yuspa S.H. (2021). RAS Induced Senescence of Skin Keratinocytes Is Mediated through Rho-Associated Protein Kinase (ROCK). Mol. Carcinog..

[67] Takai Y., Sasaki T., Matozaki T. (2001). Small GTP-Binding Proteins. Physiol. Rev..

[68] Olson M.F., Marais R. (2000). Ras Protein Signalling. Semin. Immunol..

[69] Fassl A., Geng Y., Sicinski P. (2022). CDK4 and CDK6 Kinases: From Basic Science to Cancer Therapy. Science.

[70] Cobrinik D. (2005). Pocket Proteins and Cell Cycle Control. Oncogene.

[71] Malumbres M., Sotillo R., Santamaría D., Galán J., Cerezo A., Ortega S., Dubus P., Barbacid M. (2004). Mammalian Cells Cycle without the D-Type Cyclin-Dependent Kinases Cdk4 and Cdk6. Cell.

[72] Drosten M., Dhawahir A., Sum E.Y.M., Urosevic J., Lechuga C.G., Esteban L.M., Castellano E., Guerra C., Santos E., Barbacid M. (2010). Genetic Analysis of Ras Signalling Pathways in Cell Proliferation, Migration and Survival. EMBO J..

[73] Downward J. (2003). Targeting RAS Signalling Pathways in Cancer Therapy. Nat. Rev. Cancer.

[74] Goel S., Bergholz J.S., Zhao J.J. (2022). Targeting CDK4 and CDK6 in Cancer. Nat. Rev. Cancer.

[75] Serrano M., Lin A.W., McCurrach M.E., Beach D., Lowe S.W. (1997). Oncogenic Ras Provokes Premature Cell Senescence Associated with Accumulation of P53 and p16INK4a. Cell.

[76] Jeyabalan N., Clement J.P. (2016). SYNGAP1: Mind the Gap. Front. Cell. Neurosci..

[77] Wu M., Funahashi Y., Takano T., Hossen E., Ahammad R.U., Tsuboi D., Amano M., Yamada K., Kaibuchi K. (2022). Rho–Rho-Kinase Regulates Ras-ERK Signaling Through SynGAP1 for Dendritic Spine Morphology. Neurochem. Res..

[78] Wang Q., Liu H., Wang Q., Zhou F., Liu Y., Zhang Y., Ding H., Yuan M., Li F., Chen Y. (2017). Involvement of C-Fos in Cell Proliferation, Migration, and Invasion in Osteosarcoma Cells Accompanied by Altered Expression of Wnt2 and Fzd9. PLoS ONE.

[79] McCabe L.R., Kockx M., Lian J., Stein J., Stein G. (1995). Selective Expression of Fos- and Jun-Related Genes during Osteoblast Proliferation and Differentiation. Exp. Cell Res..

[80] Angel P., Karin M. (1991). The Role of Jun, Fos and the AP-1 Complex in Cell-Proliferation and Transformation. Biochim. Biophys. Acta (BBA)-Rev. Cancer.

[81] Na H.-H., Noh H.-J., Cheong H.-M., Kang Y., Kim K.-C. (2016). SETDB1 Mediated FosB Expression Increases the Cell Proliferation Rate during Anticancer Drug Therapy. BMB Rep..

[82] Mohan H.M., Aherne C.M., Rogers A.C., Baird A.W., Winter D.C., Murphy E.P. (2012). Molecular Pathways: The Role of NR4A Orphan Nuclear Receptors in Cancer. Clin. Cancer Res..

[83] Zhao Y., Bruemmer D. (2009). NR4A Orphan Nuclear Receptors in Cardiovascular Biology. Drug Discov. Today Dis. Mech..

[84] Nomiyama T., Nakamachi T., Gizard F., Heywood E.B., Jones K.L., Ohkura N., Kawamori R., Conneely O.M., Bruemmer D. (2006). The NR4A Orphan Nuclear Receptor NOR1 Is Induced by Platelet-Derived Growth Factor and Mediates Vascular Smooth Muscle Cell Proliferation. J. Biol. Chem..

[85] Beard J.A., Tenga A., Chen T. (2015). The Interplay of NR4A Receptors and the Oncogene–Tumor Suppressor Networks in Cancer. Cell Signal..

[86] Herring J.A., Elison W.S., Tessem J.S. (2019). Function of Nr4a Orphan Nuclear Receptors in Proliferation, Apoptosis and Fuel Utilization Across Tissues. Cells.

[87] Biesiada E., Razandi M., Levin E.R. (1996). Egr-1 Activates Basic Fibroblast Growth Factor Transcription: Mechanistic Implications for Astrocyte Proliferation. J. Biol. Chem..

[88] Mayer S.I., Rössler O.G., Endo T., Charnay P., Thiel G. (2009). Epidermal-Growth-Factor-Induced Proliferation of Astrocytes Requires Egr Transcription Factors. J. Cell Sci..

[89] Sun T., Tian H., Feng Y.-G., Zhu Y.-Q., Zhang W.-Q. (2013). Egr-1 Promotes Cell Proliferation and Invasion by Increasing β-Catenin Expression in Gastric Cancer. Dig. Dis. Sci..

[90] Santiago F.S., Atkins D.G., Khachigian L.M. (1999). Vascular Smooth Muscle Cell Proliferation and Regrowth after Mechanical Injury in Vitro Are. Egr-1/NGFI-A-Dependent. Am. J. Pathol..

